# ^68^Ga-Labeled [Leu^13^ψThz^14^]Bombesin(7–14) Derivatives: Promising GRPR-Targeting PET Tracers with Low Pancreas Uptake

**DOI:** 10.3390/molecules27123777

**Published:** 2022-06-11

**Authors:** Lei Wang, Zhengxing Zhang, Helen Merkens, Jutta Zeisler, Chengcheng Zhang, Aron Roxin, Ruiyan Tan, François Bénard, Kuo-Shyan Lin

**Affiliations:** 1 Department of Molecular Oncology, BC Cancer, Vancouver, BC V5Z 1L3, Canada; lewang@bccrc.ca (L.W.); zzhang@bccrc.ca (Z.Z.); hmerkens@bccrc.ca (H.M.); jzeisler@bccrc.ca (J.Z.); cczhang@bccrc.ca (C.Z.); aroxin@bccrc.ca (A.R.); ruiyan.tan@mail.utoronto.ca (R.T.); 2 Department of Radiology, University of British Columbia, Vancouver, BC V5Z 1M9, Canada; 3 Department of Functional Imaging, BC Cancer, Vancouver, BC V5Z 4E6, Canada

**Keywords:** gastrin-releasing peptide receptor, positron-emission tomography, gallium-68, pancreas uptake

## Abstract

The gastrin-releasing peptide receptor (GRPR) is a G-protein-coupled receptor that is overexpressed in many solid cancers and is a promising target for cancer imaging and therapy. However, high pancreas uptake is a major concern in the application of reported GRPR-targeting radiopharmaceuticals, particularly for targeted radioligand therapy. To lower pancreas uptake, we explored Ga-complexed TacsBOMB2, TacsBOMB3, TacsBOMB4, TacsBOMB5, and TacsBOMB6 derived from a potent GRPR antagonist sequence, [Leu^13^ψThz^14^]Bombesin(7–14), and compared their potential for cancer imaging with [^68^Ga]Ga-RM2. The K_i_(GRPR) values of Ga-TacsBOMB2, Ga-TacsBOMB3, Ga-TacsBOMB4, Ga-TacsBOMB5, Ga-TacsBOMB6, and Ga-RM2 were 7.08 ± 0.65, 4.29 ± 0.46, 458 ± 38.6, 6.09 ± 0.95, 5.12 ± 0.57, and 1.51 ± 0.24 nM, respectively. [^68^Ga]Ga-TacsBOMB2, [^68^Ga]Ga-TacsBOMB3, [^68^Ga]Ga-TacsBOMB5, [^68^Ga]Ga-TacsBOMB6, and [^68^Ga]Ga-RM2 clearly show PC-3 tumor xenografts in positron emission tomography (PET) images, while [^68^Ga]Ga-TacsBOMB5 shows the highest tumor uptake (15.7 ± 2.17 %ID/g) among them. Most importantly, the pancreas uptake values of [^68^Ga]Ga-TacsBOMB2 (2.81 ± 0.78 %ID/g), [^68^Ga]Ga-TacsBOMB3 (7.26 ± 1.00 %ID/g), [^68^Ga]Ga-TacsBOMB5 (1.98 ± 0.10 %ID/g), and [^68^Ga]Ga-TacsBOMB6 (6.50 ± 0.36 %ID/g) were much lower than the value of [^68^Ga]Ga-RM2 (41.9 ± 10.1 %ID/g). Among the tested [Leu^13^ψThz^14^]Bombesin(7–14) derivatives, [^68^Ga]Ga-TacsBOMB5 has the highest tumor uptake and tumor-to-background contrast ratios, which is promising for clinical translation to detect GRPR-expressing tumors. Due to the low pancreas uptake of its derivatives, [Leu^13^ψThz^14^]Bombesin(7–14) represents a promising pharmacophore for the design of GRPR-targeting radiopharmaceuticals, especially for targeted radioligand therapy application.

## 1. Introduction

As a member of the G-protein-coupled receptors, the gastrin-releasing peptide receptor (GRPR) is expressed and regulates many physiological functions in the central nervous system, gastrointestinal tract, pancreas, and adrenal cortex tissues, and others [1]. Moreover, GRPR is also overexpressed in several malignancies, including melanoma, prostate, breast, and lung cancers [2,3,4,5,6,7,8]. GRPR is coupled with phospholipase C, followed by protein kinase C (PKC) activation, which regulates cell cycle, cell proliferation, and is implicated in the development of malignant neoplasms [1]. GRPR is also associated with the growth of human prostate carcinoma and pancreatic cancer by an autocrine loop with gastrin-releasing peptide (GRP) [9,10]. The overexpression of GRPR in malignant tissues has prompted development in GRPR-targeting radiopharmaceuticals for better management of GRPR-expressing cancers [11,12,13,14,15,16,17,18,19]. To date, several radiolabeled GRPR-targeting ligands (i.e., AMBA, RM2, and NeoBOMB1), based on the amphibian GRP analog bombesin, have been introduced into the clinic for cancer diagnosis and radioligand therapy [11,12,13,14,15,16]. However, high accumulations of reported GRPR-targeting radiopharmaceuticals in normal organs, particularly in the pancreas, were found in patients and preclinical animal models [11,15,16,17]. The high uptake of GRPR-targeting radiopharmaceuticals in the pancreas not only affects lesion detection, but also limits the maximum tolerated dose of targeted radioligand therapy application. 

The Schally group reported a series of GRPR antagonists based on the Bombesin(7–14) sequence that replaced Met^14^ with Thz^14^ (thiazoline-4-carboxylic acid) and introduced a reduced peptide bond (CH_2_-N) between residues 13–14 (Leu^13^ψThz^14^) [20,21]. Some of these antagonists show very potent GRPR binding affinities (low pM), and the ability to inhibit the growth of several preclinical tumor models [22,23,24]. On the basis of these studies, our group attempted to develop GRPR-targeting tracers based on the reported antagonist sequences containing a reduced peptide bond. The ^68^Ga-labeled ProBOMB1, with a p-aminomethylaniline-diglycolic acid (pABzA-DIG) linker between the DOTA chelator and D-Phe-[Leu^13^ψPro^14^]Bombesin(7–14) sequence, shows a comparable uptake in PC-3 tumors but much less uptake in normal organs/tissues than the clinically validated [^68^Ga]Ga-NeoBOMB1, especially in the pancreas (4.68 ± 1.26 vs 123 ± 28.4 %ID/g at 1 h post-injection) [25]. Replacing the pABzA-DIG linker in [^68^Ga]Ga-ProBOMB1 with 4-amino-(1-carboxymethyl)piperidine (Pip) generated [^68^Ga]Ga-ProBOMB2 which retained a good uptake in PC-3 tumors but with a reduced uptake in normal organs/tissues, especially in the pancreas and intestines, leading to further improvements in imaging contrast [25,26].

**Figure 1:**
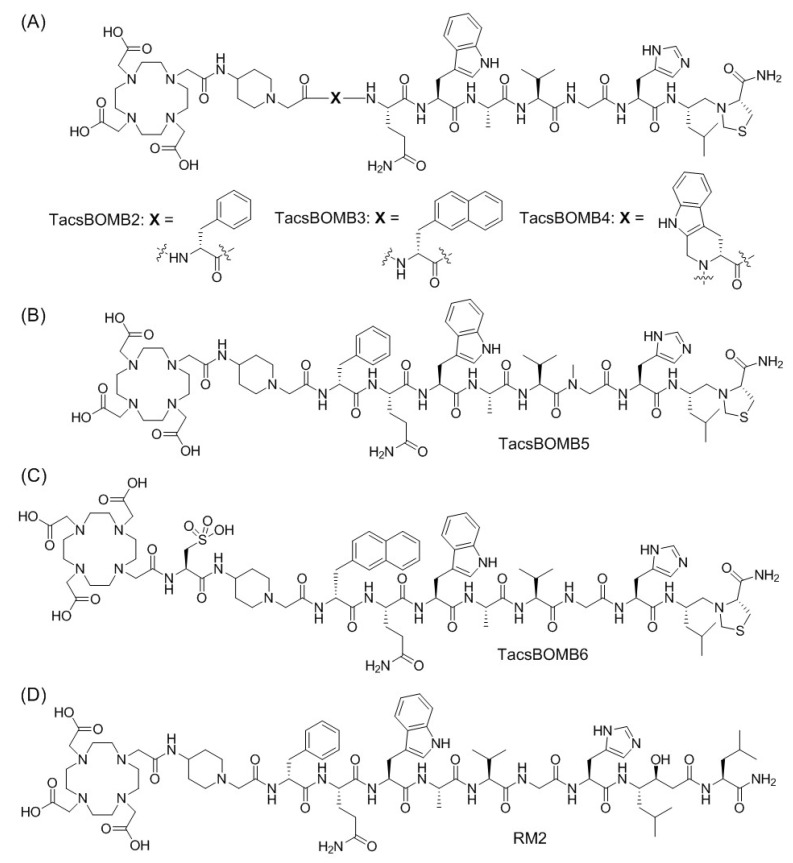
Chemical structures of (**A**) TacsBOMB2, TacsBOMB3, and TacsBOMB4, (**B**) TacsBOMB5, (**C**) TacsBOMB6, and (**D**) RM2.

ProBOMB1 and ProBOMB2 were derived from the RC-3950-II (D-Phe-[Leu^13^ψThz^14^]Bombesin(7–14)) sequence by replacing the C-terminal Thz with Pro. In this study, we synthesized Ga-complexed D-Phe-[Leu^13^ψThz^14^]Bombesin(7–14)-derived TacsBOMB2, TacsBOMB3, TacsBOMB4, TacsBOMB5, and TacsBOMB6 (Figure 1) with an unmodified C-terminal Leu^13^ψThz^14^-NH_2_. Their antagonistic characteristics were investigated using an in vitro fluorescence-based calcium-release assay. Their potential for imaging GRPR expression was evaluated by in vitro competition binding, positron emission tomography (PET) imaging, and ex vivo biodistribution studies in a preclinical PC-3 prostate cancer model in mice, and compared with the gold standard, [^68^Ga]Ga-RM2.

## 2. Results

### 2.1. Binding Affinity, Antagonist Characterization and Hydrophilicity

**Figure 2:**
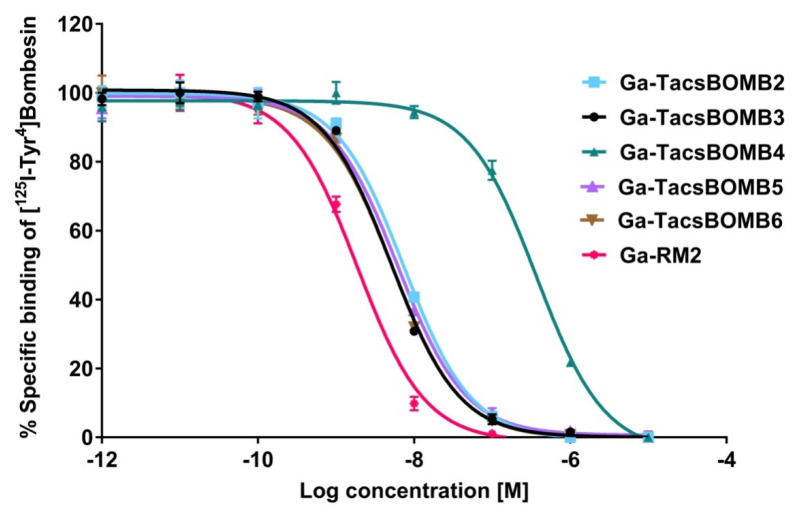
Displacement curves of [^125^I-Tyr^4^]Bombesin by Ga-TacsBOMB2, Ga-TacsBOMB3, Ga-TacsBOMB4, Ga-TacsBOMB5, Ga-TacsBOMB6, and Ga-RM2 generated using GRPR-expressing PC-3 cells. Error bars indicate standard deviation.

The binding affinities of Ga-TacsBOMB2, Ga-TacsBOMB3, Ga-TacsBOMB4, Ga-TacsBOMB5, Ga-TacsBOMB6, and Ga-RM2 were measured by a cell-based binding assay using GRPR-expressing PC-3 prostate cancer cells. Ga-TacsBOMB2, Ga-TacsBOMB3, Ga-TacsBOMB4, Ga-TacsBOMB5, Ga-TacsBOMB6, and Ga-RM2 inhibited the binding of [^125^I-Tyr^4^]Bombesin to PC-3 cells in a dose-dependent manner (Figure 2). The calculated K_i_ values for Ga-TacsBOMB2, Ga-TacsBOMB3, Ga-TacsBOMB4, Ga-TacsBOMB5, Ga-TacsBOMB6, and Ga-RM2 were 7.08 ± 0.65, 4.29 ± 0.46, 458 ± 38.6, 6.09 ± 0.95, 5.12 ± 0.57, and 1.51 ± 0.24 nM, respectively (*n* = 3).

**Figure 3:**
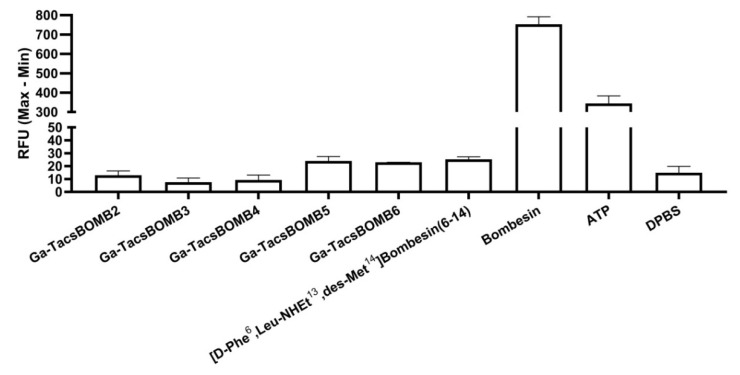
Intracellular calcium efflux in PC-3 cells induced by various tested ligands. Error bars indicate standard deviation.

The antagonistic characteristics of Ga-TacsBOMB2, Ga-TacsBOMB3, Ga-TacsBOMB4, Ga-TacsBOMB5, and Ga-TacsBOMB6 were confirmed via intracellular calcium release assays using PC-3 cells (Figure 3). ATP (50 nM) and bombesin (50 nM) as positive controls induced Ca^2+^ efflux corresponding to 334 ± 39.0 and 754 ± 38.3 relative fluorescence units (RFU), respectively, compared with 14.9 ± 4.93 RFU for the blank control, Dulbecco’s phosphate-buffered saline (DPBS). For 50 nM of Ga-TacsBOMB2, Ga-TacsBOMB3, Ga-TacsBOMB4, Ga-TacsBOMB5, and Ga-TacsBOMB6, values of 12.9 ± 3.33, 7.57 ± 3.17, 9.30 ± 3.74, 24.0 ± 3.43, 23.0 ± 0.06 RFU were observed, respectively, which were not higher than 25.3 ± 1.92 RFU obtained from the antagonist control, [D-Phe^6^,Leu-NHEt^13^,des-Met^14^]Bombesin(6–14) (50 nM).

The LogD_7.4_ values of [^68^Ga]Ga-TacsBOMB2, [^68^Ga]Ga-TacsBOMB3, [^68^Ga]Ga-TacsBOMB5, [^68^Ga]Ga-TacsBOMB6, and [^68^Ga]Ga-RM2 were −2.39 ± 0.13, −1.75 ± 0.04, −2.52 ± 0.05, −2.55 ± 0.16, and −2.76 ± 0.03, respectively (*n* = 3).

### 2.2. PET Imaging and Ex Vivo Biodistribution

**Figure 4:**
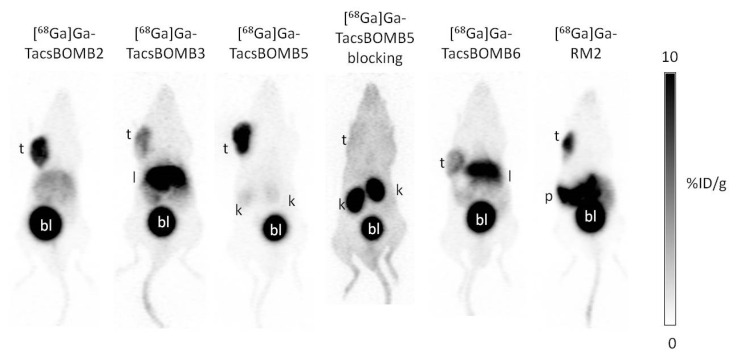
Representative maximum intensity projection PET images of [^68^Ga]Ga-TacsBOMB2, [^68^Ga]Ga-TacsBOMB3, [^68^Ga]Ga-TacsBOMB5, [^68^Ga]Ga-TacsBOMB6, and [^68^Ga]Ga-RM2 acquired at 1 h post-injection in mice bearing PC-3 tumor xenografts. t: tumor; l: liver; k: kidney; p: pancreas; bl: urinary bladder.

The PC-3 tumor xenografts were clearly visualized in PET images acquired at 1 h post-injection using [^68^Ga]Ga-TacsBOMB2, [^68^Ga]Ga-TacsBOMB3, [^68^Ga]Ga-TacsBOMB5, [^68^Ga]Ga-TacsBOMB6, and [^68^Ga]Ga-RM2 (Figure 4). All of [^68^Ga]Ga-TacsBOMB2, [^68^Ga]Ga-TacsBOMB3, [^68^Ga]Ga-TacsBOMB5, [^68^Ga]Ga-TacsBOMB6, and [^68^Ga]Ga-RM2 were primarily excreted through the renal pathway. [^68^Ga]Ga-TacsBOMB3 has a higher liver uptake than [^68^Ga]Ga-TacsBOMB2, [^68^Ga]Ga-TacsBOMB5, [^68^Ga]Ga-TacsBOMB6, and [^68^Ga]Ga-RM2. With cysteic acid as part of the linker, [^68^Ga]Ga-TacsBOMB6 has a lower liver uptake than [^68^Ga]Ga-TacsBOMB3, but its liver uptake is still higher than [^68^Ga]Ga-TacsBOMB2, [^68^Ga]Ga-TacsBOMB5, and [^68^Ga]Ga-RM2. A very high pancreas uptake was observed for [^68^Ga]Ga-RM2, but not for [^68^Ga]Ga-TacsBOMB2, [^68^Ga]Ga-TacsBOMB3, [^68^Ga]Ga-TacsBOMB5, or [^68^Ga]Ga-TacsBOMB6. Co-injection with 100 μg of nonradioactive Ga-TacsBOMB5 increased the overall background level of [^68^Ga]Ga-TacsBOMB5, especially the uptake in kidneys, and made the PC-3 tumor xenograft almost indistinguishable from the surrounding tissues.

**Figure 5:**
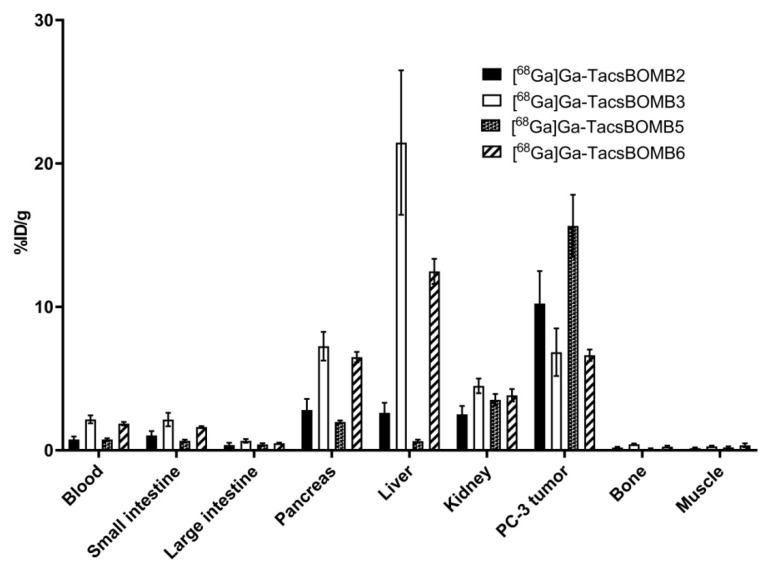
Uptake of [^68^Ga]Ga-TacsBOMB2, [^68^Ga]Ga-TacsBOMB3, [^68^Ga]Ga-TacsBOMB5, and [^68^Ga]Ga-TacsBOMB6 in PC-3 tumor xenografts and major organs/tissues of mice at 1 h post-injection. Error bars indicate standard deviation.

**Figure 6:**
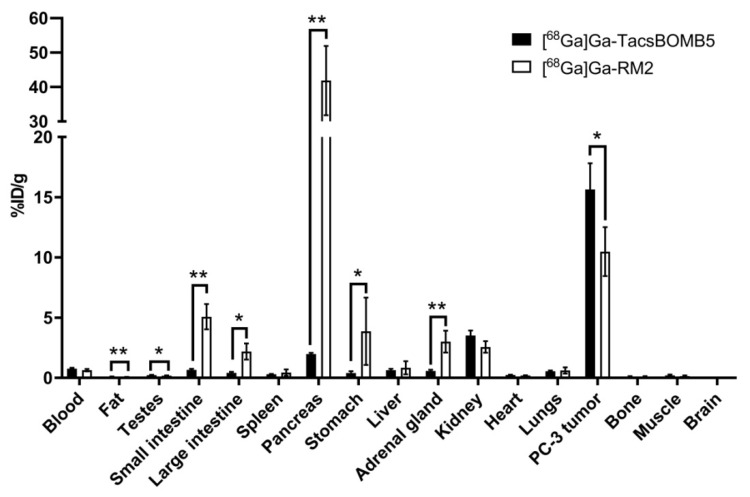
Comparison of ^68^Ga-TacsBOMB5 and ^68^Ga-RM2 uptake in PC-3 tumor xenografts and major organs/tissues in mice at 1 h post-injection. Error bars indicate standard deviation. * *p* < 0.05, ** *p* < 0.01.

**Figure 7:**
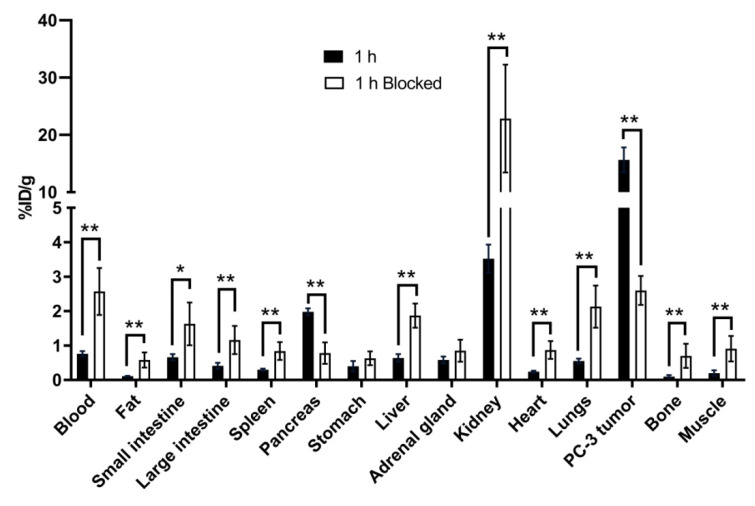
Comparison of [^68^Ga]Ga-TacsBOMB5 with/without co-injection of nonradioactive standard on the uptake in PC-3 tumor xenografts and major organs/tissues in mice at 1 h post-injection. Error bars indicate standard deviation. * *p* < 0.05, ** *p* < 0.01.

Biodistribution studies were conducted at 1 h post-injection with ^68^Ga-labeled TacsBOMB2, TacsBOMB3, TacsBOMB5, TacsBOMB6, and RM2 in PC-3 tumor-bearing mice. The results are provided in Figure 5, Figure 6 and Figure 7 and Table S4, and are consistent with the observations from their PET images. Tumor uptake values for [^68^Ga]Ga-TacsBOMB2, [^68^Ga]Ga-TacsBOMB3, [^68^Ga]Ga-TacsBOMB5, [^68^Ga]Ga-TacsBOMB6, and [^68^Ga]Ga-RM2 were 10.2 ± 2.27, 6.84 ± 1.66, 15.7 ± 2.17, 6.63 ± 0.40, and 10.5 ± 2.03 %ID/g, respectively. Pancreas uptake values for [^68^Ga]Ga-TacsBOMB2, [^68^Ga]Ga-TacsBOMB3, [^68^Ga]Ga-TacsBOMB5, [^68^Ga]Ga-TacsBOMB6, and [^68^Ga]Ga-RM2 were 2.81 ± 0.78, 7.26 ± 1.00, 1.98 ± 0.10, 6.50 ± 0.36, and 41.9 ± 10.1 %ID/g, respectively. [^68^Ga]Ga-TacsBOMB3 and [^68^Ga]Ga-TacsBOMB6 had higher liver uptake values (21.5 ± 5.04 and 12.5 ± 0.88 %ID/g, respectively) than the other tracers ([^68^Ga]Ga-TacsBOMB2: 2.61 ± 0.70 %ID/g; [^68^Ga]Ga-TacsBOMB5: 0.64 ± 0.11 %ID/g; [^68^Ga]Ga-RM2: 0.84 ± 0.55 %ID/g). Uptake values of brain, muscle, bone, heart, and spleen were <1% ID/g for all evaluated tracers.

Compared with [^68^Ga]Ga-RM2, [^68^Ga]Ga-TacsBOMB5 has a significantly higher tumor uptake but a lower uptake in most major organs, especially in the pancreas, leading to higher tumor-to-organ (bone, muscle, blood, kidney, and pancreas) uptake ratios (Figure 6 and Table S4).

Co-injection of nonradioactive Ga-TacsBOMB5 reduces the average uptake of [^68^Ga]Ga-TacsBOMB5 in the PC-3 tumor xenografts by 83% (15.7 down to 2.60 %ID/g at 1 h post-injection), confirming its specific uptake in tumors. In addition, a significant reduction in the average uptake of [^68^Ga]Ga-TacsBOMB5 was also found in the pancreas (1.98 down to 0.78 %ID/g at 1 h post-injection), which indicates its specific uptake in the pancreas. On the contrary, the average uptake values of [^68^Ga]Ga-TacsBOMB5 in other major organs were increased at 1 h post-injection with the co-injection of nonradioactive Ga-TacsBOMB5 (Figure 7 and Table S4).

### 2.3. In Vivo Stability

Both [^68^Ga]Ga-TacsBOMB2 and [^68^Ga]Ga-TacsBOMB5 were shown to be sufficiently stable in vivo in NRG mice (*n* = 3), with 83.3 ± 1.45% and 67.1 ± 4.76%, respectively, remaining intact in plasma at 15 min post-injection (Figures S1 and S2). These values are not significantly different from the intact fraction of [^68^Ga]Ga-RM2 (71.9 ± 10.4%, Figure S3). On the other hand, no intact [^68^Ga]Ga-TacsBOMB2, [^68^Ga]Ga-TacsBOMB5, or [^68^Ga]Ga-RM2 were detected in the mouse urine samples collected at 15 min post-injection (Figures S1–S3).

## 3. Discussion

To the best of our knowledge, this is the first report on the development of GRPR-targeting tracers based on the [Leu^13^ψThz^14^]Bombesin(7–14) sequence. The designs of TacsBOMB2, TacsBOMB3, and TacsBOMB4 (Figure 1) are based on the potent GRPR antagonists reported by the Schally group: RC-3950-II (D-Phe-[Leu^13^ψThz^14^]Bombesin(7–14)); RC-3965-II (D-2-Nal-[Leu^13^ψThz^14^]Bombesin(7–14)); and RC-3910-II ((D-Tpi-[Leu^13^ψThz^14^]Bombesin(7–14)), respectively [20,21]. TacsBOMB5 is an NMe-Gly^11^ derivative of TacsBOMB2 as replacing Gly^11^ with NMe-Gly was previously reported to improve the metabolic stability of GRPR-targeting tracers [27,28]. TacsBOMB6, with the addition of a cysteic acid between the Pip linker and the DOTA chelator, was designed to improve the hydrophilicity of TacsBOMB3. During our first attempt, the amino acids, Pip linker, and DOTA chelator were sequentially coupled to the Rink Amide MBHA resin for the synthesis of TacsBOMB2. However, after treating the resin with trifluoroacetic acid for cleavage, followed by precipitation with diethyl ether, very little crude product was obtained (data not shown). After checking with MS analysis, the major peak of the isolated crude product showed a molecular weight of ~300 dalton higher than the expected product of TacsBOMB2 (see Figures S4 and S5). This is consistent with the observation by Yraola et al. [29], and it is likely due to the protonation of the reduced peptide bond (a tertiary amine) between Leu^13^ and Thz^14^ by trifluoroacetic acid, which strengthened the amide bond between Thz^14^ and the Rink Amide MBHA resin. Therefore, instead of cleavage at the C-N bond where Thz^14^ was coupled to the Rink Amide MBHA resin, a labile C-N bond on the resin component was cleaved instead (Figure S4). To fix this problem, a more acid-labile Sieber resin was used, and the desired TacsBOMB2, TacsBOMB3, TacsBOMB4, TacsBOMB5, and TacsBOMB6 were successfully isolated and characterized (Table S1).

The average K_i_ values of Ga-TacsBOMB2, Ga-TacsBOMB3, Ga-TacsBOMB5, and Ga-TacsBOMB6 are comparable (4.29–7.08 nM). This suggests that replacing D-Phe in Ga-TacsBOMB2 with D-2-Nal to obtain Ga-TacsBOMB3, replacing Gly^11^ in Ga-TacsBOMB2 with NMe-Gly to obtain Ga-TacsBOMB5, or the addition of a cysteic acid between the Pip linker and DOTA chelator of Ga-TacsBOMB3 to obtain Ga-TacsBOMB6 does not have a major effect on their GRPR-binding affinity. However, replacing D-Phe in Ga-TacsBOMB2 with D-Tpi to obtain Ga-TacsBOMB4 results in a significant loss of binding affinity (K_i_ = 7.08 ± 0.65 vs. 458 ± 38.6 nM). This is likely due to the rigidity of the secondary amino group of D-Tpi, which prohibits free rotation of the added Ga-DOTA complex and Pip linker; therefore, this affects its binding to GRPR.

Calcium release assays revealed that compared to the antagonist control, [D-Phe^6^,Leu-NHEt^13^,des-Met^14^]Bombesin(6-14) (50 nM), there was no higher calcium efflux observed by 50 nM of Ga-TacsBOMB2, Ga-TacsBOMB3, Ga-TacsBOMB4, Ga-TacsBOMB5, and Ga-TacsBOMB6, confirming their antagonistic characteristics. This suggests that the addition of the Ga-DOTA complex and Pip linker to the N-terminus of the reported GRPR antagonists RC-3950-II (Ga-TacsBOMB2), RC-3965-II (Ga-TacsBOMB3) and RC-3910-II (Ga-TacsBOMB4) does not change their antagonistic characteristics. Similarly, replacing Gly^11^ with NMe-Gly (Ga-TacsBOMB5), or the addition of a cysteic acid between the Pip linker and DOTA chelator (Ga-TacsBOMB6), does not change their antagonistic characteristics either.

LogD_7.4_ measurements confirmed the hydrophilic properties of [^68^Ga]Ga-TacsBOMB2, [^68^Ga]Ga-TacsBOMB3, [^68^Ga]Ga-TacsBOMB4, [^68^Ga]Ga-TacsBOMB5, and [^68^Ga]Ga-TacsBOMB6. Replacing D-Phe in [^68^Ga]Ga-TacsBOMB2 with a bulkier D-2-Nal in [^68^Ga]Ga-TacsBOMB3 increased the lipophilicity (LogD_7.4_ = −2.39 ± 0.13 vs. −1.75 ± 0.04). The addition of a cysteic acid to [^68^Ga]Ga-TacsBOMB3 resulted in a more hydrophilic [^68^Ga]Ga-TacsBOMB6 (LogD_7.4_ = −1.75 ± 0.04 vs. −2.55 ± 0.16), as expected. The reduction in the average LogD_7.4_ values from [^68^Ga]Ga-TacsBOMB2 (−2.39 ± 0.13) to [^68^Ga]Ga-TacsBOMB5 (−2.52 ± 0.05) was unexpected as Gly in [^68^Ga]Ga-TacsBOMB2 is less lipophilic than NMe-Gly in [^68^Ga]Ga-TacsBOMB5. This suggests that the change in LogD_7.4_ value cannot be determined by an individual replaced amino acid but has to take into account the interaction of the replaced amino acid with the remaining components of the peptide.

PET imaging and biodistribution studies (Figure 4 and Figure 5 and Table S4) confirm the good GRPR-targeting capabilities of [^68^Ga]Ga-TacsBOMB2, [^68^Ga]Ga-TacsBOMB3, [^68^Ga]Ga-TacsBOMB5, and [^68^Ga]Ga-TacsBOMB6, as PC-3 tumors were clearly visualized in their PET images. Compared to [^68^Ga]Ga-TacsBOMB2, [^68^Ga]Ga-TacsBOMB3 has a lower tumor uptake (10.2 ± 2.27 vs. 6.84 ± 1.66 %ID/g), likely due to its more lipophilic nature, which also results in a higher liver uptake (2.61 ± 0.70 vs. 21.5 ± 5.04). The addition of a cysteic acid lowered the liver uptake of [^68^Ga]Ga-TacsBOMB6 (12.5 ± 0.88 %ID/g), but no improvement in tumor uptake (6.63 ± 0.40 %ID/g) was observed. Replacing Gly^11^ in [^68^Ga]Ga-TacsBOMB2 with NMe-Gly resulted in [^68^Ga]Ga-TacsBOMB5, which caused a 54% increase in tumor uptake (10.2 ± 2.27 vs. 15.7 ± 2.17 %ID/g) and superior tumor-to-background contrast ratios.

To demonstrate the potential for clinical translation of [^68^Ga]Ga-TacsBOMB5 to detect GRPR-expressing cancers, we conducted head-to-head comparisons with the clinically validated [^68^Ga]Ga-RM2. As shown in Figure 4, Figure 5 and Figure 6 and Table S4, compared to [^68^Ga]Ga-RM2, [^68^Ga]Ga-TacsBOMB5 has a higher tumor uptake (10.5 ± 2.03 vs. 15.7 ± 2.17 %ID/g), a much lower pancreas uptake (41.9 ± 10.1 vs. 1.98 ± 0.10 %ID/g) and higher tumor-to-normal organ uptake ratios, especially the tumor-to-pancreas ratio (0.25 ± 0.04 vs. 7.95 ± 1.40). The relatively lower average pancreas uptake (1.98–7.26 %ID/g, Table S4) of [^68^Ga]Ga-TacsBOMB2, [^68^Ga]Ga-TacsBOMB3, [^68^Ga]Ga-TacsBOMB5, and [^68^Ga]Ga-TacsBOMB6 is consistent with the observation from [^68^Ga]Ga-ProBOMB1 and [^68^Ga]Ga-ProBOMB2 derived from D-Phe-[Leu^13^ψPro^14^]Bombesin(7–14) (25–26). Our data suggest that D-Phe-[Leu^13^ψPro^14^]Bombesin(7–14) and D-Phe-[Leu^13^ψThz^14^]Bombesin(7–14) are promising peptide sequences for the design of GRPR-targeting radiopharmaceuticals with a low pancreas uptake, especially for radioligand therapy applications to minimize toxicity to the pancreas.

In vivo stability studies were conducted to investigate if the higher tumor uptake of [^68^Ga]Ga-TacsBOMB5 compared to [^68^Ga]Ga-RM2 was the result of improved stability from the NMe-Gly replacement. As shown in Figures S2 and S3, [^68^Ga]Ga-TacsBOMB5 was not more stable in vivo than [^68^Ga]Ga-RM2 against peptidase degradation, as their intact fractions in plasma at 15 min post-injection were 67.1 ± 4.76 and 71.9 ± 10.4%, respectively. In addition, the GRPR-binding affinity of [^68^Ga]Ga-TacsBOMB5 was not better than [^68^Ga]Ga-RM2 either, as their K_i_ values were 5.12 ± 0.57 and 1.51 ± 0.24 nM, respectively. One possible explanation is the much lower uptake of [^68^Ga]Ga-TacsBOMB5 in the pancreas when compared to [^68^Ga]Ga-RM2 (1.98 ± 0.10 vs. 41.9 ± 10.1 %ID/g), enabling more chances for the circulating [^68^Ga]Ga-TacsBOMB5 to bind to GRPR in PC-3 tumors.

The blocking study (Figure 7 and Table S4) shows that the average uptake of [^68^Ga]Ga-TacsBOMB5 in PC-3 tumors is reduced by 83% with the co-injection of nonradioactive standard, confirming its specific uptake in tumors. In addition, the average uptake of [^68^Ga]Ga-TacsBOMB5 in the pancreas is also reduced by 60%, suggesting there is also some specific uptake of [^68^Ga]Ga-TacsBOMB5 in the pancreas. This is in agreement with the observation that the pancreas is probably the highest GRPR-expressing normal organ [1,2,3]. However, compared to the clinically validated [^68^Ga]Ga-RM2 and [^68^Ga]Ga-NeoBOMB1, [^68^Ga]Ga-TacsBOMB5 has ~50% more uptake in PC-3 tumors (10.5 ± 2.03 and 9.83 ± 1.48 %ID/g, respectively vs. 15.7 ± 2.17 %ID/g), but only a small fraction of uptake in the mouse pancreas (41.9 ± 10.1 and 122 ± 28.4 %ID/g, respectively vs. 1.98 ± 0.10 %ID/g). This suggests that the extremely high uptake of [^68^Ga]Ga-RM2 and [^68^Ga]Ga-NeoBOMB1 might not be entirely mediated by GRPR but possibly by some other off-targets as well. However, it cannot rule out the possibility that, compared to [^68^Ga]Ga-RM2 and [^68^Ga]Ga-NeoBOMB1, [^68^Ga]Ga-TacsBOMB5 might be more selective for the human GRPR expressed in PC-3 tumors compared to the mouse GRPR expressed in the mouse pancreas. Further clinical studies of [^68^Ga]Ga-TacsBOMB5 are needed to validate if the observations from the mouse model can be translated to humans.

## 4. Materials and Methods

### 4.1. Synthesis of GRPR-Targeting Ligands

Detailed information for the synthesis and purification of TacsBOMB2, TacsBOMB3, TacsBOMB4, TacsBOMB5, and TacsBOMB6, their nonradioactive Ga-complexed standards, and ^68^Ga-labeled derivatives is provided in the supplementary file (Scheme S1 and Tables S1–S3).

### 4.2. LogD_7.4_ Measurement

LogD_7.4_ values of [^68^Ga]Ga-TacsBOMB2, [^68^Ga]Ga-TacsBOMB3, [^68^Ga]Ga-TacsBOMB5, [^68^Ga]Ga-TacsBOMB6, and [^68^Ga]Ga-RM2 were measured using the shake–flask method as previously published [30]. Briefly, aliquots (2 μL) of the ^68^Ga-labeled peptides were added to a vial containing 3 mL of 1-octanol and 3 mL of 0.1 M phosphate buffer (pH 7.4). The mixture was vortexed for 1 min and then centrifuged at 5000 rpm for 10 min. Samples of the 1-octanol (1 mL) and buffer (1 mL) layers were taken and counted in a gamma counter. LogD_7.4_ was calculated using the following equation: LogD_7.4_ = log_10_[(counts in 1-octanol phase)/(counts in buffer phase)].

### 4.3. Cell Culture

The PC-3 cells obtained from ATCC (via Cedarlane, Burlington, Canada) were cultured in RPMI 1640 medium (Life Technologies Corporations) supplemented with 10% FBS, penicillin (100 U/mL), and streptomycin (100 μg/mL) at 37 °C in a Panasonic Healthcare (Tokyo, Japan) MCO-19AIC humidified incubator containing 5% CO_2_. The IMPACT Rodent Pathogen Test (IDEXX BioAnalytics) verified that the cells were pathogen-free. Cells were washed with sterile phosphate-buffered saline (PBS, pH 7.4) and collected after 1 min trypsinization when grown to 80–90% confluence. The cell concentration was counted in triplicate using a hemocytometer and a manual laboratory counter.

### 4.4. Fluorometric Calcium Release Assay

Following previously published procedures [25,26], 5 × 10^4^ PC-3 cells were seeded in 96-well clear-bottom black plates 24 h prior to the assay. The growth medium was removed and replaced with a loading buffer containing a calcium-sensitive dye (FLIPR Calcium 6 assay kit, Molecular Device, San Jose, CA, USA). After incubation at 37 °C for 30 min, the plates were placed in a FlexStation 3 microplate reader (Molecular Devices). Ga-TacsBOMB2 (50 nM), Ga-TacsBOMB3 (50 nM), Ga-TacsBOMB4 (50 nM), Ga-TacsBOMB5 (50 nM), Ga-TacsBOMB6 (50 nM), [D-Phe^6^,Leu-NHEt^13^,des-Met^14^]Bombesin(6–14) (50 nM, antagonist control), bombesin (50 nM, positive control), adenosine triphosphate (ATP, 50 nM, positive control), or Dulbecco’s phosphate-buffered saline (DPBS) were added to the cells, and the fluorescent signals were acquired for 2 min (λ_Ex_ = 485 nm; λ_Em_ = 525 nm; *n* = 2). Agonistic/antagonistic properties were determined using the relative fluorescent unit (RFU = max–min).

### 4.5. In Vitro Competition Binding Assay

PC-3 cells were seeded at 2 × 10^5^ cells/well in 24-well poly-D-lysine plates 24–48 h prior to the experiment. The growth medium was replaced by 400 μL of reaction medium (RPMI 1640 containing 2 mg/mL BSA and 20 mM HEPES). Cells were incubated for 30–60 min at 37 °C. Ga-TacsBOMB2, Ga-TacsBOMB3, Ga-TacsBOMB4, Ga-TacsBOMB5, Ga-TacsBOMB6, or Ga-RM2 in 50 μL of decreasing concentrations (10 μM to 1 pM) and 50 μL of 0.011 nM [^125^I-Tyr^4^]Bombesin (Perkin Elmer, Waltham, MA) were added to wells, followed by incubation with moderate agitation for 1 h at 27 °C. Cells were gently washed with ice-cold PBS twice, harvested by trypsinization, and measured for radioactivity on a Perkin Elmer (Waltham, MA, USA) Wizard2 2480 automatic gamma counter. Data were analyzed using nonlinear regression (one binding site model for competition assay) with GraphPad (San Diego, CA, USA) Prism 8 software.

### 4.6. Ex Vivo Biodistribution, PET/CT Imaging and In Vivo Stability Studies

Imaging, biodistribution, and in vivo stability studies were performed using male NOD.Cg-Rag1^tm1Mom^ Il2rg^tm1Wjl^/SzJ (NRG) mice (from in-house breeding colonies) following previously published procedures [25,26,30,31]. The experiments were conducted according to the guidelines established by the Canadian Council on Animal Care and approved by the Animal Ethics Committee of the University of British Columbia. The mice were anesthetized by inhalation of 2.5% isoflurane in oxygen and implanted subcutaneously with 5 × 10^6^ PC-3 cells (100 µL; 1:1 PBS/Matrigel) behind the left shoulder. When the tumor grew to 5–8 mm in diameter over 3–4 weeks, the mice were used for PET/CT imaging and biodistribution studies.

The PET/CT imaging experiments were carried out using a Siemens (Knoxville, TN, USA) Inveon micro-PET/CT scanner. Each tumor-bearing mouse (*n* = 1–2) was injected with ~3–6 MBq (0.05–0.1 nmol) of a ^68^Ga-labeled tracer through a lateral caudal tail vein under 2.5% isoflurane in oxygen anesthesia, followed by a recovery period in which it could roam freely in its cage during the uptake period. At 50 min post-injection, a 10 min CT scan was conducted: first for localization and attenuation corrections after segmentation for reconstructing the PET images, followed by a 10 min static PET imaging acquisition.

For biodistribution studies, the mice (*n* = 4) were injected with the radiotracer (~2–4 MBq, 0.03–0.07 nmol) as described above. For blocking, the mice were co-injected with 100 μg of nonradioactive Ga-TacsBOMB5. At 1 h post-injection, the mice were euthanized by CO_2_ inhalation. Blood was withdrawn by cardiac puncture, and organs/tissues of interest were collected, weighed and counted using a Perkin Elmer (Waltham, MA, USA) Wizard2 2480 automatic gamma counter.

For in vivo stability studies, [^68^Ga]Ga-TacsBOMB2 (6.10 ± 0.04 MBq), [^68^Ga]Ga-TacsBOMB5 (9.60 ± 0.56 MBq), and [^68^Ga]Ga-RM2 (5.56 ± 0.03 MBq) were injected via the lateral caudal vein into healthy male NRG mice (*n* = 3). At 15 min post-injection, the mice were sedated and euthanized, and urine and blood were collected. The plasma was extracted from whole blood by the addition of CH_3_CN (500 μL), vortexing, centrifugation, and the separation of the supernatants. The plasma and urine samples were analyzed via radio-HPLC by using the conditions for quality control of these ^68^Ga-labeled radioligands (Table S3).

### 4.7. Statistical Analysis

Statistical analyses were performed by Student’s *t*-test using the Microsoft (Redmond, WA, USA) Excel software. The unpaired two-tailed test was used to compare biodistribution data of [^68^Ga]Ga-TacsBOMB5 and [^68^Ga]Ga-RM2. The unpaired one-tailed test was used to compare the biodistribution data of [^68^Ga]Ga-TacsBOMB5 with/without co-injection of nonradioactive Ga-TacsBOMB5. A statistically significant difference was considered when the adjusted *p* value was <0.05.

## 5. Conclusions

Modifications on the reported potent GRPR antagonists, D-Phe-[Leu^13^ψThz^14^]Bombesin(7–14) and D-2-Nal-[Leu^13^ψThz^14^]Bombesin(7–14), do not affect their GRPR-targeting capability. The resulting ^68^Ga-labeled TacsBOMB2, TacsBOMB3, TacsBOMB5, and TacsBOMB6 clearly visualize GRPR-expressing PC-3 tumors in PET images. Among them, [^68^Ga]Ga-TacsBOMB5 shows a superior tumor uptake and tumor-to-background contrast ratios compared to the clinically validated [^68^Ga]Ga-RM2. Most importantly, the pancreas uptake of [^68^Ga]Ga-TacsBOMB5 is only a small fraction (~5%) of that of [^68^Ga]Ga-RM2. Consequently, [^68^Ga]Ga-TacsBOMB5 should be more sensitive for detecting GRPR-expressing pancreatic cancer and other cancer lesions adjacent to the pancreas. Due to the low pancreas uptake of ^68^Ga-labeled tracers derived from D-Phe-[Leu^13^ψThz^14^]Bombesin(7–14) and D-2-Nal-[Leu^13^ψThz^14^]Bombesin(7–14), these two peptide sequences are promising for the design of GRPR-targeting radiopharmaceuticals, especially for radioligand therapy application to minimize toxicity to the pancreas.

## 6. Patents

The compounds disclosed in this report are covered by a recent patent application (PCT/CA2019/051620). Zhengxing Zhang, Jutta Zeisler, François Bénard and Kuo-Shyan Lin are listed as inventors of this filed patent.

## Data Availability

The data presented in this study are available in the Supplementary Materials.

## Supplementary Materials

The following supporting information can be downloaded at: https://www.mdpi.com/article/10.3390/molecules27123777/s1. Detailed synthetic procedures and results for the preparation of TacsBOMB2, TacsBOMB3, TacsBOMB4, TacsBOMB5, TacsBOMB6, and their ^nat^Ga/^68^Ga-complexed analogs; Scheme S1: Synthesis of Fmoc-LeuψThz-OH hydrochloride (**3**); Table S1: HPLC purification conditions and MS characterizations of TacsBOMB2, TacsBOMB3, TacsBOMB4, TacsBOMB5, and TacsBOMB6; Table S2: HPLC purification conditions and MS characterizations of Ga-TacsBOMB2, Ga-TacsBOMB3, Ga-TacsBOMB4, Ga-TacsBOMB5, and Ga-TacsBOMB6; Table S3: HPLC conditions for the purification and quality control of ^68^Ga-labeled TacsBOMB2, TacsBOMB3, TacsBOMB5, and TacsBOMB6; Table S4: Biodistribution (mean ± SD, *n* = 4) and uptake ratios of ^68^Ga-labeled GRPR-targeting tracers in PC-3 tumor-bearing mice; Figure S1: Representative radio-HPLC chromatograms from analysis of intact fraction of [^68^Ga]Ga-TacsBOMB2 in mouse plasma (a) and urine (b) samples collected at 15 min post-injection; Figure S2: Representative radio-HPLC chromatograms from analysis of intact fraction of [^68^Ga]Ga-TacsBOMB5 in mouse plasma (a) and urine (b) samples collected at 15 min post-injection; Figure S3: Representative radio-HPLC chromatograms from analysis of intact fraction of [^68^Ga]Ga-RM2 in mouse plasma (a) and urine (b) samples collected at 15 min post-injection; Figure S4: The proposed chemical structure of the observed by-product from cleavage of protected TacsBOMB2 off the Rink Amide MBHA resin; Figure S5: MS analysis of the observed by-product [30,32,33,34].

## References

[1] Jensen R., Battey J., Spindel E., Benya R. (2008). International Union of Pharmacology. LXVIII. Mammalian bombesin receptors: Nomenclature, distribution, pharmacology, signaling, and functions in normal and disease states. Pharmacol. Rev..

[2] Cornelio D.B., Roesler R., Schwartsmann G. (2007). Gastrin-releasing peptide receptor as a molecular target in experimental anticancer therapy. Ann. Oncol..

[3] Hajri A., Koenig M., Balboni G., Damgé C. (1996). Expression and characterization of gastrin-releasing peptide receptor in normal and cancerous pancreas. Pancreas.

[4] Moody T., Zia F., Venugopal R., Fagarasan M., Oie H., Hu V. (1996). GRP receptors are present in non small cell lung cancer cells. J. Cell Biochem..

[5] Preston S., Woodhouse L., Jones-Blackett S., Miller G., Primrose J. (1995). High-affinity binding sites for gastrin-releasing peptide on human colorectal cancer tissue but not uninvolved mucosa. Br. J. Cancer.

[6] Gugger M., Reubi J.C. (1999). Gastrin-releasing peptide receptors in non-neoplastic and neoplastic human breast. Am. J. Pathol..

[7] Preston S.R., Woodhouse L.F., Gokhale J., Miller G.V., Primrose J.N. (1994). Characterization of a bombesin/gastrin-releasing peptide receptor on a human gastric-cancer cell line. Int. J. Cancer.

[8] Markwalder R., Reubi J.C. (1999). Gastrin-releasing peptide receptors in the human prostate: Relation to neoplastic transformation. Cancer Res..

[9] Shimoda J. (1992). Effects of bombesin and its antibody on growth of human prostatic carcinoma cell lines. Jpn. J. Urol..

[10] Qin Y., Ertl T., Cai R.-Z., Halmos G., Schally A.V. (1994). Inhibitory effect of bombesin receptor antagonist RC-3095 on the growth of human pancreatic cancer cells in vivo and in vitro. Cancer Res..

[11] Baum R., Prasad V., Mutloka N., Frischknecht M., Maecke H., Reubi J. (2007). Molecular imaging of bombesin receptors in various tumors by Ga-68 AMBA PET/CT: First results. J. Nucl. Med..

[12] Kähkönen E., Jambor I., Kemppainen J., Lehtiö K., Grönroos T.J., Kuisma A., Luoto P., Sipilä H.J., Tolvanen T., Alanen K. (2013). In vivo imaging of prostate cancer using [^68^Ga]-labeled bombesin analog BAY86-7548. Clin. Cancer Res..

[13] Stoykow C., Erbes T., Maecke H.R., Bulla S., Bartholomä M., Mayer S., Drendel V., Bronsert P., Werner M., Gitsch G. (2016). Gastrin-releasing peptide receptor imaging in breast cancer using the receptor antagonist ^68^Ga-RM2 and PET. Theranostics.

[14] Baratto L., Song H., Duan H., Hatami N., Bagshaw H., Buyyounouski M., Hancock S., Shah S.A., Srinivas S., Swift P. (2021). PSMA- and GRPR-targeted PET: Results from 50 patients with biochemically recurrent prostate cancer. J. Nucl. Med..

[15] Kurth J., Krause B.J., Schwarzenböck S.M., Bergner C., Hakenberg O.W., Heuschkel M. (2020). First-in-human dosimetry of gastrin-releasing peptide receptor antagonist [^177^Lu]Lu-RM2: A radiopharmaceutical for the treatment of metastatic castration-resistant prostate cancer. Eur. J. Nucl. Med. Mol. Imaging.

[16] Nock B.A., Kaloudi A., Lymperis E., Giarika A., Kulkarni H.R., Klette I., Singh A., Krenning E.P., de Jong M., Maina T. (2017). Theranostic perspectives in prostate cancer with the gastrin-releasing peptide receptor antagonist NeoBOMB1: Preclinical and first clinical results. J. Nucl. Med..

[17] Minamimoto R., Hancock S., Schneider B., Chin F.T., Jamali M., Loening A., Vasanawala S., Gambhir S.S., Iagaru A. (2016). Pilot comparison of ^68^Ga-RM2 PET and ^68^Ga-PSMA-11 PET in patients with biochemically recurrent prostate cancer. J. Nucl. Med..

[18] Gasser G., Tjioe L., Graham B., Belousoff M.J., Juran S., Walther M., Künstler J., Bergmann R., Stephan H., Spiccia L. (2008). Synthesis, copper(II) complexation, ^64^Cu-labeling, and bioconjugation of a new bis(2-pyridylmethyl) derivative of 1,4,7-triazacyclononane. Bioconjugate Chem..

[19] Juran S., Walther M., Stephan H., Bergmann R., Steinbach J., Kraus W., Emmerling F., Comba P. (2009). Hexadentate bispidine derivatives as versatile bifunctional chelate agents for copper(II) radioisotopes. Bioconjugate Chem..

[20] Reile H., Cai R., Armatis P., Schally A. (1995). New antagonists of bombesin gastrin-releasing peptide with C-terminal Leuψ(CH_2_N)Tac-NH_2_. Int. J. Oncol..

[21] Cai R., Reile H., Armatis P., Schally A.V. (1994). Potent bombesin antagonists with C-terminal Leu-ψ(CH_2_-N)-Tac-NH_2_ or its derivatives. Proc. Natl. Acad. Sci. USA.

[22] Jungwirth A., Pinski J., Galvan G., Halmos G.B., Szepeshazi K.R., Gai R., Groot K., Vadillo-Buenfil M., Schally A.V. (1997). Inhibition of growth of androgen-independent DU-145 prostate cancer in vivo by luteinising hormone-releasing hormone antagonist Cetrorelix and bombesin antagonists RC-3940-II and RC-3950-II. Eur. J. Cancer.

[23] Koppán M., Halmos G., Arencibia J.M., Lamharzi N., Schally A.V. (1998). Bombesin/gastrin-releasing peptide antagonists RC-3095 and RC-3940-II inhibit tumor growth and decrease the levels and mRNA expression of epidermal growth factor receptors in H-69 small cell lung carcinoma. Cancer.

[24] Shirahige Y., Cai R.-Z., Szepeshazi K., Halmos G., Pinski J., Groot K., Schally A. (1994). Inhibitory effect of bombesin/gastrin-releasing peptide (GRP) antagonists RC-3950-II and RC-3095 on MCF-7 MIII human breast cancer xenografts in nude mice. Biomed. Pharmacother..

[25] Lau J., Rousseau E., Zhang Z., Uribe C.F., Kuo H.-T., Zeisler J., Zhang C., Kwon D., Lin K.-S., Bénard F. (2019). Positron emission tomography imaging of the gastrin-releasing peptide receptor with a novel bombesin analogue. ACS Omega.

[26] Bratanovic I.J., Zhang C., Zhang Z., Kuo H.T., Colpo N., Zeisler J., Merkens H., Uribe C., Lin K.S., Bénard F. (2022). A Radiotracer for molecular imaging and therapy of gastrin-releasing peptide receptor–positive prostate cancer. J. Nucl. Med..

[27] Höhne A., Mu L., Honer M., Schubiger P.A., Ametamey S.M., Graham K., Stellfeld T., Borkowski S., Berndorff D., Klar U. (2008). Synthesis, ^18^F-Labeling, and in vitro and in vivo studies of bombesin peptides modified with silicon-based building blocks. Bioconjugate Chem..

[28] Richter S., Wuest M., Bergman C.N., Krieger S., Rogers B.E., Wuest F. (2016). Metabolically stabilized ^68^Ga-NOTA-Bombesin for PET imaging of prostate cancer and influence of protease inhibitor phosphoramidon. Mol. Pharm..

[29] Yraola F., Ventura R., Vendrell M., Colombo A., Fernàndez J.C., de la Figuera N., Fernández-Forner D., Royo M., Forns P., Albericio F. (2004). A Re-evaluation of the Use of Rink, BAL, and PAL Resins and Linkers. QSAR Comb. Sci..

[30] Lin K.-S., Pan J., Amouroux G., Turashvili G., Mesak F., Hundal-Jabal N., Pourghiasian M., Lau J., Jenni S., Aparicio S. (2015). In vivo radioimaging of bradykinin receptor B1, a widely overexpressed molecule in human cancer. Cancer Res..

[31] Kuo H.-T., Pan J., Zhang Z., Lau J., Merkens H., Zhang C., Colpo N., Lin K.-S., Benard F. (2018). Effects of linker modification on tumor-to-kidney contrast of ^68^Ga-labeled PSMA-targeted imaging probes. Mol. Pharm..

[32] Lodhi M.A., Shams S., Khan K.M. (2014). Thiazolidine esters: New potent urease inhibitors. J. Chem. Soc. Pak..

[33] Amouroux G., Pan J., Jenni S., Zhang C., Zhang Z., Hundal-Jabal N., Colpo N., Liu Z., Bénard F., Lin K.-S. (2015). Imaging human bradykinin B1 receptor with ^68^Ga-labeled [des-Arg^10^]kallidin derivatives: Effect of the linker on biodistribution and tumor uptake. Mol. Pharm..

[34] Lin K.-S., Amouroux G., Pan J., Zhang Z., Jenni S., Lau J., Hundal-Jabal N., Colpo N., Benard F. (2015). Comparative studies of three gallium-68-labeled [des-Arg10]kallidin derivatives for imaging bradykinin B1 receptor expression with positron emission tomography. J. Nucl. Med..

