# Supplementary material for: 68Ga-Labeled [Leu13ψThz14]Bombesin(7–14) Derivatives: Promising GRPR-Targeting PET Tracers with Low Pancreas Uptake
Source: Molecules. 2022 Jun 11;27(12):3777. doi: 10.3390/molecules27123777 (PMC9230575; doi:10.3390/molecules27123777)
Supplement: Supplementary file 1 [file molecules-27-03777-s001.zip › molecules-1744898-supplementary.pdf]

**SUPPLEMENTAL INFORMATION**

## General Methods

(4*R*)-*t*-Butyl 4-thiazolidinecarboxylate hydrochloride (**1**) was synthesized following literature procedures (*1*). All other chemicals and solvents were purchased from commercial sources and used without further purification. GRPR-targeting peptides were synthesized using a solid phase approach with an AAPPTec (Louisville, KY) Endeavor 90 peptide synthesizer. Purification and quality control of DOTA-conjugated peptides and their $^{nat}$Ga/$^{68}$Ga-complexed analogs were performed on Agilent (Santa Clara, CA) HPLC systems equipped with a model 1200 quaternary pump, a model 1200 UV absorbance detector (220 nm), and a Bioscan (Washington, DC) NaI scintillation detector. The operation of Agilent HPLC systems was controlled using the Agilent ChemStation software. The HPLC columns used were a semi-preparative column (Luna C18, 5 µm, 250 × 10 mm) and an analytical column (Luna C18, 5 µm, 250 × 4.6 mm) purchased from Phenomenex (Torrance, CA). The collected HPLC eluates were lyophilized using a Labconco (Kansas City, MO) FreeZone 4.5 Plus freeze-drier. MS analyses were conducted using the Waters (Milford, MA) Acquity QDa mass spectrometer with the equipped 2489 UV/Vis detector and e2695 Separations module. C18 Sep-Pak cartridges (1 cm$^3$, 50 mg) were purchased from Waters (Milford, MA). $^{68}$Ga was eluted from an iThemba Labs (Somerset West, South Africa) generator, and purified according to the previously published procedures using a DGA resin column from Eichrom Technologies LLC (Lisle, IL) (*2*). The radioactivity of $^{68}$Ga-labeled peptides was measured using a Capintec (Ramsey, NJ) CRC®-25R/W dose calibrator and the radioactivity of mouse tissues collected from biodistribution studies were counted using a Perkin Elmer (Waltham, MA) Wizard2 2480 automatic gamma counter.

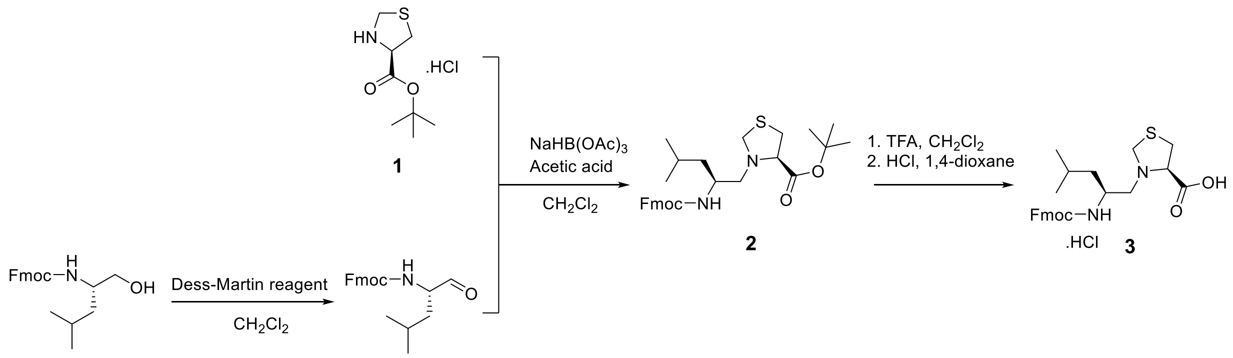


**Scheme S1**: Synthesis of Fmoc-LeuψThz-OH hydrochloride (**3**).

**Synthesis of Fmoc-LeuψThz-OH Hydrochloride (3)**

Compound **3** was synthesized following the reaction steps depicted in Scheme 1.

Synthesis of Fmoc-LeuψThz-OtBu (**2**)

Solution 1: Fmoc-Leucinol (3.79 g, 11.1 mmol) was converted to the aldehyde with Dess-Martin periodinane (5.87 g, 13.8 mol) in dichloromethane (70 mL) under ice/water bath for 4 h. The reaction mixture was then mixed with saturated $NaHCO_3$ aqueous solution (130 mL) and sodium thiosulfate (13.0 g) and stirred for 30 min before being extracted with dichloromethane (130 mL). The organic layer was collected, dried over anhydrous magnesium sulfate, concentrated in vacuo to ~ 20 mL in volume.

Solution 2: Compound **1** (1.72 g, 7.62 mmol) was dissolved in saturated $NaHCO_3$ aqueous solution (35 mL) and the mixture was extracted with ethyl acetate (100 mL × 2). The organic phases were combined, dried over anhydrous magnesium sulfate, evaporated in vacuo to obtain colorless oil. The oil was mixed with acetic acid (400 μL, 7.0 mmol) in dichloromethane (30 mL).

Solutions 1 and 2 were mixed and the mixture was stirred for 30 min at room temperature. Sodium triacetoxyborohydride (5.41 g, 25.5 mmol) was added into the mixture and stirred for 20 h. Saturated $NaHCO_3$ aqueous solution (100 mL) was added and stirred for 10 min. The mixture was extracted with ethyl acetate (100 mL × 2). The organic phases were combined, dried over anhydrous $MgSO_4$ and purified by flash column chromatography eluted with 1:3 ether/hexanes to obtain **2** as a white solid (2.13 g, 63% yield). ESI-MS: m/z calculated for $[M+H]^+$ of **2** $C_{29}H_{38}N_2O_4S$ 511.7; found 511.5. $^1H$ NMR (600 MHz, $CDCl_3$) δ 7.76 (d, $J$ = 7.5 Hz, 2H, Ar-$H$), 7.60 (t, $J$ = 6.5 Hz, 2H, Ar-$H$), 7.39 (t, $J$ = 7.4 Hz, 2H, Ar-$H$), 7.30 (t, $J$ = 7.3 Hz, 2H, Ar-$H$), 4.49 – 4.35 (m, 2H, O$CH_2$), 4.22 (t, $J$ = 6.6 Hz, 1H, $OCH_2CH$), 4.17 – 3.96 (m, 2H, S$CH_2$N), 3.93 – 3.76 (m, 2H, S$CH_2$CH), 3.18 (d, $J$ = 8.7 Hz, NHC$H$), 3.08 – 2.97 (m, 1H, COOC$H$), 2.59 – 2.43 (m, 2H, NHC$H_2$CH), 1.71-1.60 (m, 1H, $CH_3CH$), 1.45 (s, 9H), 1.40 – 1.32 (m, 2H, $CH_3CHCH_2$), 0.93 (2, 6H, C$H_3$).

Synthesis of Fmoc-LeuψThz-OH (**3**)

Compound **2** was dissolved in a mixture of dichloromethane (25 mL) and trifluoroacetic acid (75 mL), and stirred for 3 h at room temperature. After concentrated in vacuo, the residue was dissolved in ethyl acetate (80 mL) and mixed with 4M HCl in 1,4-dioxane dioxane (3 mL). After being stirred for 10 min, the volatile solvents were removed in vacuo. Diethyl ether (250 mL) was added to the residue and the mixture was stirred for 30 min. White solid was collected by filtration to obtain 1.38 g of **3** (70% yield) as a white solid. ESI-MS: m/z calculated for $[M+H]^+$ of **3** $C_{25}H_{30}N_2O_4S$ 455.6; found 455.4. $^1H$ NMR (600 MHz, MeOD) δ 7.81 (d, $J$ = 7.5 Hz, 2H, Ar-$H$), 7.68 (dd, $J$ = 10.3, 7.6 Hz, 2H, Ar-$H$), 7.41 (t, $J$ = 7.4 Hz, 2H, Ar-$H$), 7.36 – 7.30 (m, 2H, Ar-$H$), 4.73 – 4.62 (m, 1H, $OCH_2CH$), 4.53 – 4.50 (m, 2H, O$CH_2$), 4.29 – 4.21 (m, 2H, S$CH_2$N), 4.07 – 3.91 (m, 1H, NHC$H$), 3.54 – 3.39 (m, 2H, S$CH_2$CH), 3.29 – 3.22 (m, 2H, NHC$H_2$CH), 3.03 (dd, $J$ = 13.0, 9.6 Hz, 1H, COOHC$H$), 1.64 – 1.58 (m, 1H, $CH_3CH$), 1.46 – 1.27 (m, 2H, $CH_3CHCH_2$), 0.94 (dd, $J$ = 12.7, 6.6 Hz, 6H, C$H_3$).

## Synthesis of DOTA-conjugated Peptides

TacsBOMB2, TacsBOMB3, TacsBOMB4, and TacsBOMB5 were synthesized on solid phase using Fmoc peptide chemistry. Sieber resin (0.05 mmol, 0.104g) was treated with 20% piperidine in *N*,*N*-dimethylformamide (DMF) to remove Fmoc protecting group. After removal of the Fmoc protecting group, Fmoc-LeuψThz-OH (**3**) (3 eq.), Fmoc-protected amino acids (5 eq.), Fmoc-4-amino-(1-carboxymethyl)piperidine (5 eq.) were pre-activated with HATU (3 eq.), HOAt (3 eq.), and *N*,*N*-diisopropylethylamine (DIEA, 9 eq.) before being sequentially coupled to the resin. Then DOTA(*t*Bu)$_3$ (5 eq.) pre-activated with HATU (5 eq.) and DIEA (25 eq.) was coupled to the resin. TacsBOMB6 was synthesized following similar procedures with the addition of Fmoc-cysteic acid before the coupling of DOTA($^t$Bu)$_3$.

The peptides were deprotected and simultaneously cleaved from the resin with a mixture of trifluoroacetic acid (TFA, 81.5%), triisopropylsilane (TIS 1.0%), water (5%), 2,2′-(ethylenedioxy)diethanethiol (DODT, 2.5%), thioanisole (5%), and phenol (5%) for 4 h at room temperature. The cleaved peptides were filtrated and then precipitated by the addition of cold diethyl ether. The crude peptides were collected by centrifugation and purified with HPLC (semi-preparative column; flow rate: 4.5 mL/min). The eluates containing the desired peptides were collected and lyophilized. The HPLC conditions, retention times, isolated yields and MS confirmations of DOTA-conjugated peptides are provided in the Supplemental Table 1.

## Synthesis of Nonradioactive Ga-complexed Standards

The nonradioactive Ga-complexed standards were prepared using a solution of the DOTA-conjugated precursor mixed and incubated with $GaCl_3$ (5 eq.) in NaOAc buffer (0.1 M, 500 µL, pH 4.2 – 4.5) at 80 °C for 15 min. The reaction mixture was then purified via HPLC (semi-preparative column, flow rate: 4.5 mL/min). The HPLC eluates containing the desired peptide were collected and lyophilized. The HPLC conditions, retention times, isolated yields and MS confirmations of these nonradioactive Ga-complexed standards are provided in the Supplemental Table 2.

## Synthesis of $^{68}$Ga-labeled Compounds

The radiolabeling experiments were performed following previously published procedures (*2-4*). Purified $^{68}GaCl_3$ in 0.5 mL water was added to a 4-mL glass vial preloaded with 0.7 mL of HEPES buffer (2 M, pH 5.0) and 10 µL precursor solution (1 mM). The radiolabeling reaction was carried out under microwave heating for 1 min before being purified by HPLC using the semi-preparative column. The eluate fraction containing the radiolabeled product was collected, diluted with water (50 mL), and passed through a C18 Sep-Pak cartridge that was pre-washed with ethanol (10 mL) and water (10 mL). After washing the C18 Sep-Pak cartridge with water (10 mL), the $^{68}$Ga-labeled product was eluted off the cartridge with ethanol (0.4 mL), and diluted with PBS for imaging and biodistribution studies. Quality control was performed using the analytical column. The HPLC conditions and retention times are provided in the Supplemental Table 3. The tracers were obtained in 42-59% decay-corrected radiochemical yields with >66 GB/µmol molar activity and >92% radiochemical purity.

**Supplemental Table S1:** HPLC purification conditions and MS characterizations of TacsBOMB2, TacsBOMB3, TacsBOMB4, TacsBOMB5 and TacsBOMB6.

| Compound name | HPLC conditions | Retention time (min) | Yield (%) | Calculated mass (m/z) | Found (m/z) |
|---|---|---|---|---|---|
| TacsBOMB2 | 25% $CH_3CN$ and 0.1% TFA in $H_2O$ | 18.7 | 34 | $[M+2H]^{2+}$ 792.4 | $[M+2H]^{2+}$ 792.8 |
| TacsBOMB3 | 28% $CH_3CN$ and 0.1% TFA in $H_2O$ | 15.8 | 49 | $[M+2H]^{2+}$ 817.4 | $[M+2H]^{2+}$ 817.8 |
| TacsBOMB4 | 28% $CH_3CN$ and 0.1% TFA in $H_2O$ | 16.6 | 38 | $[M+2H]^{2+}$ 817.9 | $[M+2H]^{2+}$ 818.2 |
| TacsBOMB5 | 25% $CH_3CN$ and 0.1% TFA in $H_2O$ | 18.3 | 32 | $[M+2H]^{2+}$ 799.4 | $[M+2H]^{2+}$ 799.7 |
| TacsBOMB6 | 29% $CH_3CN$ and 0.1% TFA in $H_2O$ | 14.6 | 14 | $[M+2H]^{2+}$ 892.9 | $[M+2H]^{2+}$ 893.4 |

**Supplemental Table S2:** HPLC purification conditions and MS characterizations of Ga-complexed TacsBOMB2, TacsBOMB3, TacsBOMB4, TacsBOMB5 and TacsBOMB6.

| Compound name | HPLC conditions | Retention time (min) | Yield (%) | Calculated mass (m/z) | Found (m/z) |
|---|---|---|---|---|---|
| Ga-TacsBOMB2 | 26% $CH_3CN$ and 0.1% TFA in $H_2O$ | 12.5 | 72 | $[M+2H]^{2+}$ 825.9 | $[M+2H]^{2+}$ 826.0 |
| Ga-TacsBOMB3 | 28% $CH_3CN$ and 0.1% TFA in $H_2O$ | 18.3 | 67 | $[M+2H]^{2+}$ 850.9 | $[M+2H]^{2+}$ 850.7 |
| Ga-TacsBOMB4 | 28% $CH_3CN$ and 0.1% TFA in $H_2O$ | 18.8 | 68 | $[M+2H]^{2+}$ 851.4 | $[M+2H]^{2+}$ 851.2 |
| Ga-TacsBOMB5 | 25% $CH_3CN$ and 0.1% TFA in $H_2O$ | 18.3 | 75 | $[M+2H]^{2+}$ 832.9 | $[M+2H]^{2+}$ 832.8 |
| Ga-TacsBOMB6 | 29% $CH_3CN$ and 0.1% TFA in $H_2O$ | 14.6 | 57 | $[M+2H]^{2+}$ 926.4 | $[M+2H]^{2+}$ 926.4 |

**Supplemental Table S3:** HPLC conditions for the purification and quality control of $^{68}$Ga-labeled TacsBOMB2, TacsBOMB3, TacsBOMB5, and TacsBOMB6. FA: formic acid.

| Compound name | HPLC conditions | | Retention time (min) |
|---|---|---|---|
| [$^{68}$Ga]Ga-TacsBOMB2 | Semi-Prep | 20% $CH_3CN$ and 0.1% FA in $H_2O$; flow rate 4.5 mL/min | 12.6 |
| | QC | 23% $CH_3CN$ and 0.1% FA in $H_2O$; flow rate 2.0 mL/min | 7.2 |
| [$^{68}$Ga]Ga-TacsBOMB3 | Semi-Prep | 21% $CH_3CN$ and 0.1% FA in $H_2O$; flow rate 4.5 mL/min | 35.8 |
| | QC | 26% $CH_3CN$ and 0.1% FA in $H_2O$; flow rate 2 mL/min | 8.8 |
| [$^{68}$Ga]Ga-TacsBOMB5 | Semi-Prep | 20% $CH_3CN$ and 0.1% FA in $H_2O$; flow rate 4.5 mL/min | 10.7 |
| | QC | 23% $CH_3CN$ and 0.1% FA in $H_2O$; flow rate 2.0 mL/min | 5.1 |
| [$^{68}$Ga]Ga-TacsBOMB6 | Semi-Prep | 29% $CH_3CN$ and 0.1% TFA in $H_2O$; flow rate 4.5 mL/min | 14.6 |
| | QC | 28% $CH_3CN$ and 0.1% FA in $H_2O$; flow rate 2.0 mL/min | 6.7 |

**Supplemental Table S4:** Biodistribution (mean ±SD,n= 4) and uptake ratios of $^{68}$Ga-labeled GRPR-targeting tracers in PC-3 tumor-bearing mice. The mice in the blocked group were co-injected with 100 µg of nonradioactive Ga-TacsBOMB5.

| Tissue (%ID/g) | [$^{68}$Ga]Ga-TacsBOMB2 | [$^{68}$Ga]Ga-TacsBOMB3 | [$^{68}$Ga]Ga-TacsBOMB5 | | [$^{68}$Ga]Ga-TacsBOMB6 | [$^{68}$Ga]Ga-RM2 |
|---|---|---|---|---|---|---|
| | 1 h | 1 h | 1 h | 1 h blocked | 1 h | 1 h |
| Blood | 0.76 ± 0.21 | 2.16 ± 0.28 | 0.76 ± 0.08 | 2.57 ± 0.68 | 1.86 ± 0.12 | 0.64 ± 0.10 |
| Fat | 0.09 ± 0.03 | 0.19 ± 0.08 | 0.11 ± 0.01 | 0.58 ± 0.22 | 0.25 ± 0.05 | 0.05 ± 0.03 |
| Testes | 0.19 ± 0.05 | 0.33 ± 0.08 | 0.23 ± 0.02 | 1.06 ± 0.13 | 0.60 ± 0.16 | 0.18 ± 0.03 |
| Small intestine | 1.04 ± 0.30 | 2.15 ± 0.47 | 0.66 ± 0.09 | 1.63 ± 0.62 | 1.62 ± 0.07 | 5.08 ± 1.05 |
| Large intestine | 0.37 ± 0.16 | 0.66 ± 0.13 | 0.41 ± 0.09 | 1.16 ± 0.41 | 0.50 ± 0.04 | 2.19 ± 0.67 |
| Spleen | 0.47 ± 0.17 | 0.68 ± 0.15 | 0.30 ± 0.03 | 0.84 ± 0.26 | 0.84 ± 0.17 | 0.44 ± 0.26 |
| Pancreas | 2.81 ± 0.78 | 7.26 ± 1.00 | 1.98 ± 0.10 | 0.78 ± 0.31 | 6.50 ± 0.36 | 41.9 ± 10.1 |
| Stomach | 0.32 ± 0.08 | 1.10 ± 0.18 | 0.40 ± 0.15 | 0.63 ± 0.20 | 0.57 ± 0.07 | 3.87 ± 2.80 |
| Liver | 2.61 ± 0.70 | 21.5 ± 5.04 | 0.64 ± 0.11 | 1.87 ± 0.35 | 12.5 ± 0.88 | 0.84 ± 0.55 |
| Adrenal glands | 0.57 ± 0.40 | 1.81 ± 0.72 | 0.58 ± 0.10 | 0.85 ± 0.32 | 1.27 ± 0.33 | 3.01 ± 0.91 |
| Kidneys | 2.51 ± 0.59 | 4.49 ± 0.51 | 3.52 ± 0.41 | 22.9 ± 9.41 | 3.84 ± 0.43 | 2.57 ± 0.48 |
| Heart | 0.27 ± 0.08 | 0.66 ± 0.09 | 0.24 ± 0.03 | 0.87 ± 0.26 | 0.58 ± 0.04 | 0.19 ± 0.03 |
| Lungs | 0.75 ± 0.52 | 3.05 ± 1.29 | 0.55 ± 0.07 | 2.13 ± 0.61 | 1.95 ± 0.96 | 0.62 ± 0.26 |
| PC-3 tumor | 10.2 ± 2.27 | 6.84 ± 1.66 | 15.7 ± 2.17 | 2.60 ± 0.42 | 6.63 ± 0.40 | 10.5 ± 2.03 |
| Bone | 0.19 ± 0.06 | 0.42 ± 0.06 | 0.10 ± 0.04 | 0.70 ± 0.35 | 0.26 ± 0.07 | 0.11 ± 0.03 |
| Muscle | 0.15 ± 0.05 | 0.28 ± 0.05 | 0.20 ± 0.08 | 0.91 ± 0.37 | 0.35 ± 0.14 | 0.14 ± 0.06 |
| Brain | 0.05 ± 0.03 | 0.06 ± 0.03 | 0.03 ± 0.01 | 0.08 ± 0.02 | 0.05 ± 0.00 | 0.03 ± 0.01 |
| Tumor/bone | 61.3 ± 25.0 | 17.0 ± 5.84 | 175 ± 82.4 | 4.37 ± 1.99 | 27.5 ± 7.98 | 96.5 ± 27.1 |
| Tumor/muscle | 70.1 ± 14.2 | 26.0 ± 9.92 | 82.3 ± 19.2 | 3.21 ± 1.18 | 20.5 ± 6.45 | 80.8 ± 27.5 |
| Tumor/blood | 14.0 ± 3.48 | 3.28 ± 1.19 | 20.6 ± 2.96 | 1.05 ± 0.25 | 3.58 ± 0.24 | 16.5 ± 3.06 |
| Tumor/kidney | 4.10 ± 0.46 | 1.55 ± 0.50 | 4.48 ± 0.69 | 0.13 ± 0.05 | 1.73 ± 0.13 | 4.13 ± 0.73 |
| Tumor/pancreas | 3.70 ± 0.55 | 0.98 ± 0.37 | 7.95 ± 1.40 | 3.77 ± 1.50 | 1.02 ± 0.05 | 0.25 ± 0.04 |

**(A)**

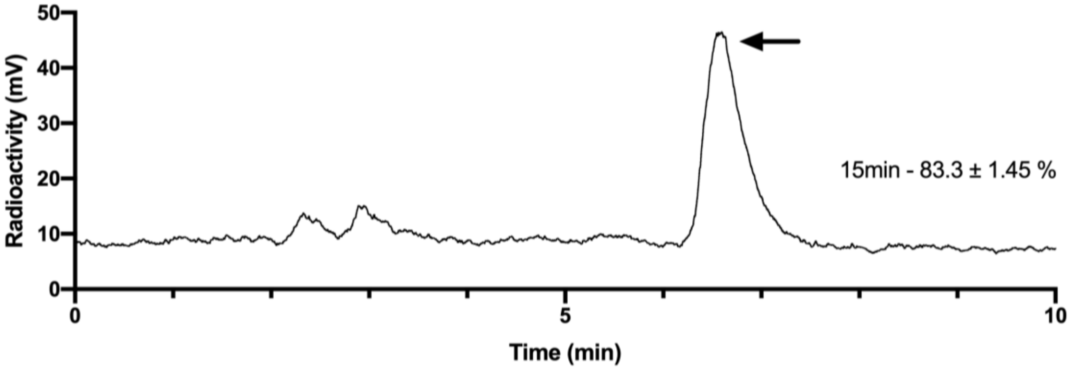


**(B)**

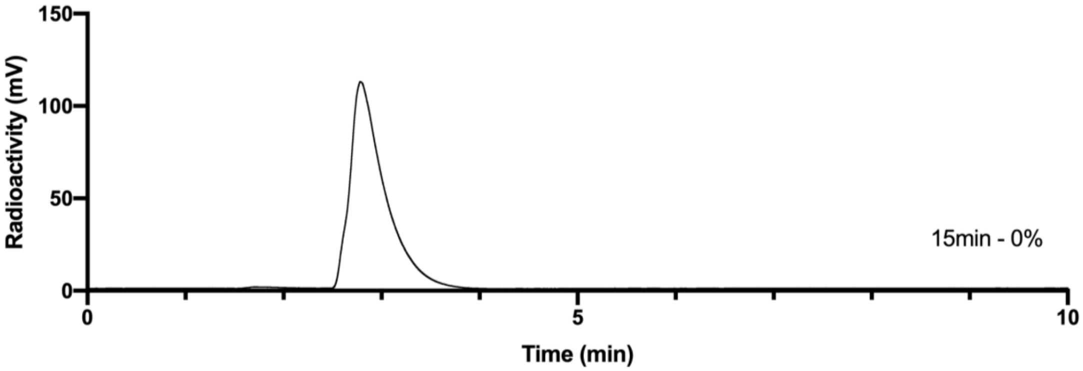


**Supplemental Figure S1.** Representative radio-HPLC chromatograms from analysis of intact fraction of [$^{68}$Ga]Ga-TacsBOMB2 in mouse plasma (A) and urine (B) samples collected at 15 min post-injection. The peak pointed by an arrow is the intact tracer.

**(A)**

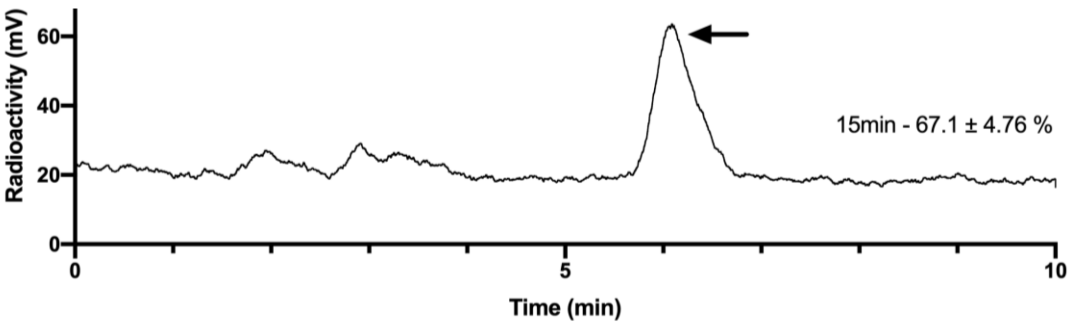


**(B)**

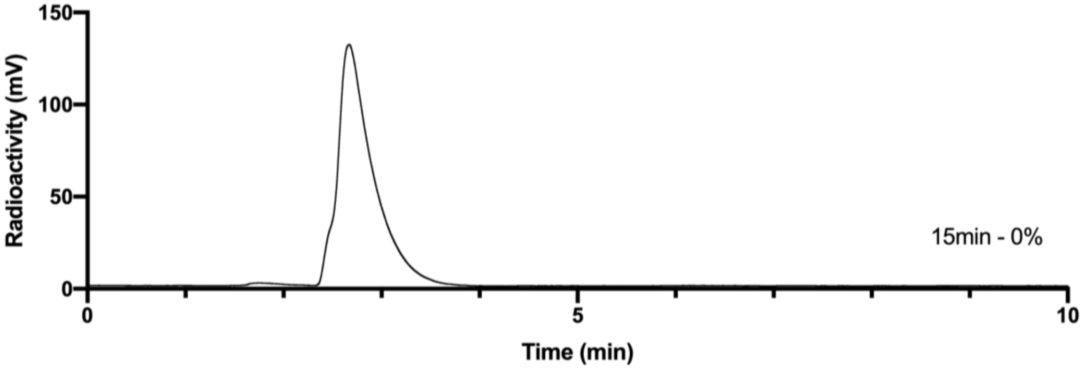


**Supplemental Figure S2.** Representative radio-HPLC chromatograms from analysis of intact fraction of [$^{68}$Ga]Ga-TacsBOMB5 in mouse plasma (A) and urine (B) samples collected at 15 min post-injection. The peak pointed by an arrow is the intact tracer.

(A)

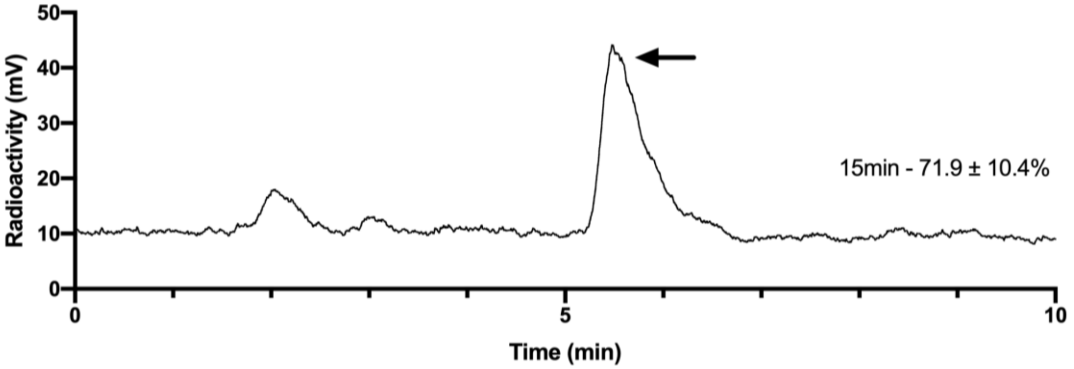


(B)

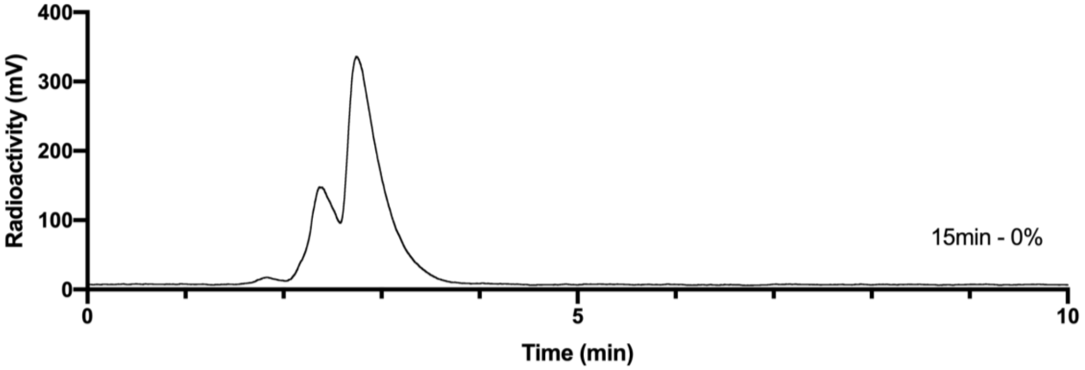


**Supplemental Figure S3.** Representative radio-HPLC chromatograms from analysis of intact fraction of [$^{68}$Ga]Ga-RM2 in mouse plasma (A) and urine (B) samples collected at 15 min post-injection. The peak pointed by an arrow is the intact tracer.

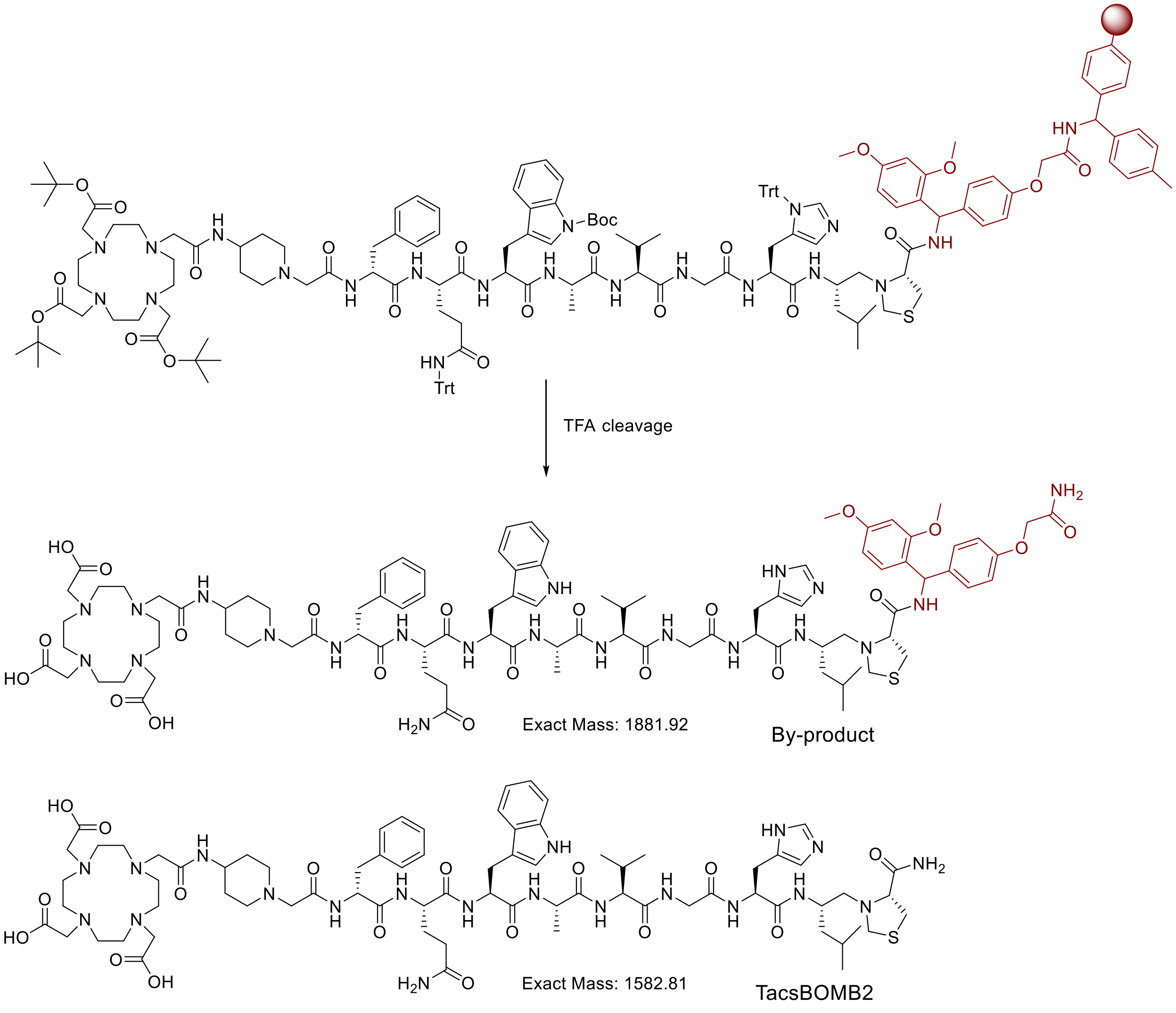


**Supplemental Figure S4.** The proposed chemical structure of the observed by-product from cleavage of protected TacsBOMB2 off the Rink Amide MBHA resin. The motif in brown comes from the cleavage of another C-N bond on the Rink Amide MBHA resin.

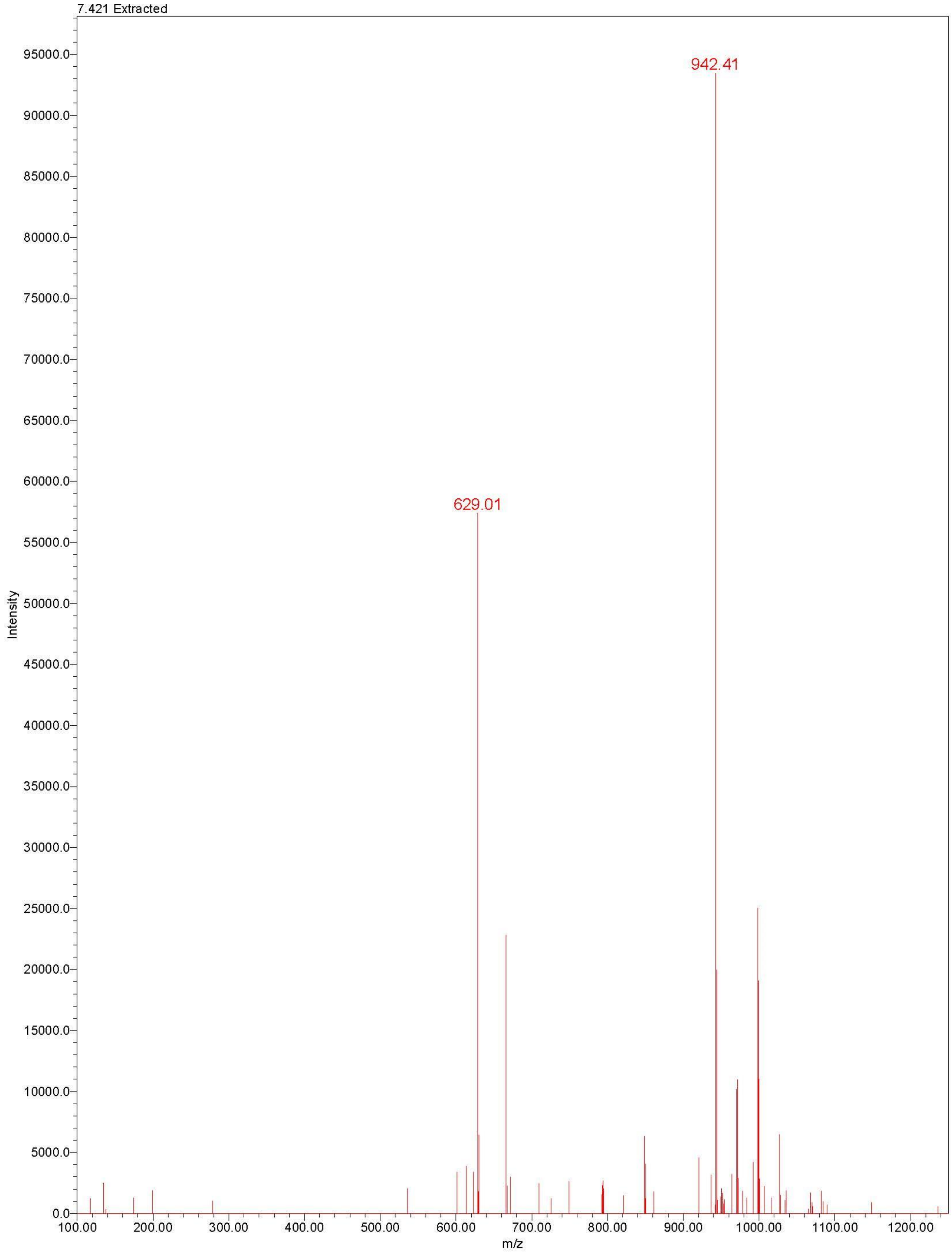


**Supplemental Figure S5.** MS analysis of the observed by-product. The 942.41 and 629.01 m/z peaks are the observed $[M+2H]^{2+}$ and $[M+3H]^{3+}$ peaks of the by-product with a calculated molecular weight of 1881.92 (see the Supplemental Figure 4).
